# Bikini line one-anastomosis gastric bypass (BLOGB): initial report

**DOI:** 10.1007/s00464-024-11242-7

**Published:** 2024-09-26

**Authors:** Tamer N. Abdelbaki, Yomna E. Dean

**Affiliations:** General Surgery Department, Faculty of Medicine, Alexandria University, 11 Hussein Nouh St, Shalalat, Bab Sharki, Alexandria, Egypt

**Keywords:** One-anastomosis gastric bypass, Bikini line, Minimal scar, Aesthetic outcomes

## Abstract

**Background:**

This study introduces a new access method for one-anastomosis gastric bypass (OAGB) by placing ports at the bikini line.

**Objective:**

To describe our initial experience and assess the feasibility, safety, and effectiveness of this novel access.

**Setting:**

University Hospital.

**Methods:**

This prospective case–control study included 72 patients: 42 were allocated to the bikini line one-anastomosis gastric bypass (BLOGB) group, and 30 were assigned to the control group. Exclusion criteria included a history of major abdominal surgery, hiatal hernia, extensive lower abdominal adhesions, or a body mass index (BMI) exceeding 55 kg/m^2^.

**Results:**

The mean preoperative BMI of the study sample was 40.01 ± 2.84. Weight loss was satisfactory, with the highest percent excess weight loss (%EWL) observed at 12 months: 90.88 ± 7.90 and 91 ± 7.11 (*p* = 0.474) in both groups, respectively. Both groups showed no significant differences in operative complications, hospital stay, weight loss, or resolution of obesity-associated diseases. However, the BLOGB patients had a longer mean operative time of 110.71 ± 17.72 min compared to 98 ± 18.27 min in the control group (*p* = 0.002). Moreover, they experienced less postoperative pain and reported greater satisfaction with the appearance of their scars.

**Conclusion:**

BLOGB was found to be potentially feasible, safe, and effective, offering improved aesthetic outcomes and reduced postoperative pain. This approach may be suitable for a select group of patients concerned with scar appearance. However, large-scale studies are necessary to ensure that safety is not compromised in pursuit of aesthetic benefits.

The global surge in obesity rates is a significant health concern, with over 1 billion individuals identified as having obesity by the World Health Organization (WHO) [1]. According to the "Guidelines for the Management of Overweight and Obesity in Adults," jointly issued by the American College of Cardiology, the American Heart Association, and the Obesity Society, 64.5% of adults with obesity are recommended to undergo weight loss treatment [2].

Metabolic/bariatric surgery is widely recognized as a highly effective intervention for **severe** obesity, demonstrating significant success in promoting weight loss and improving obesity-associated medical problems [3]. Laparoscopic sleeve gastrectomy (LSG) and laparoscopic Roux-en-Y gastric bypass (LRYGB) are currently the most commonly performed bariatric procedures worldwide [4]. However, the one-anastomosis gastric bypass (OAGB) has become increasingly prevalent in bariatric surgical procedures globally, constituting 4.6% of all surgeries performed [5, 6]. It has gained traction, particularly among patients with **class V obesity**, due to its straightforward technique, shorter operative times, and lower complication rates. Studies have shown that OAGB could lead to higher rates of resolution of obesity-associated medical problems and better quality of life superior to SG and comparable to RYGB [7–9].

Over the past decades, bariatric surgery has evolved to minimize surgical trauma and enhance aesthetic outcomes, particularly among young women and patients concerned about the appearance of their scars [10]. The lead author previously described the bikini line sleeve gastrectomy (BLSG) and the bikini line hiatal hernia repair (BLHHR) [11, 12]. In these approaches, conventional ports were placed in the lower abdomen along the bikini line, the curved line just above the symphysis pubis. These methods were found to be potentially safe, with patients reporting favorable aesthetic outcomes. Building on this, the present study introduces a new access method to minimize surgical trauma and improve aesthetic results in OAGB. Termed "Bikini Line One-Anastomosis Gastric Bypass" (BLOGB), this technique involves positioning the incision ports along the bikini line within the suprapubic region. This manuscript aims to describe our initial experience and assess the feasibility and safety of BLOGB compared to the conventional OAGB approach.

## Patients and methods

Between December 2020 and March 2023, we conducted a preliminary prospective case–control study targeting patients with obesity eligible for one-anastomosis gastric bypass who opted for the novel "Bikini line" approach, referred to as Bikini Line One-anastomosis Gastric Bypass (BLOGB). This approach was offered to all consecutive patients eligible for OAGB with a BMI less than 55 kg/m^2^. Exclusion criteria comprised prior major abdominal surgery, hiatal hernia, extensive lower abdominal adhesions detected intra-operatively, and a body mass index (BMI) exceeding 55 kg/m^2^. The exclusion of individuals with a high BMI in this initial investigation aimed to enhance safety and ensure technical success.

During the study period, 72 consecutive patients were admitted to undergo OAGB, with forty-two opting for BLOGB. All patients were followed up for at least 6 months, and informed consent was obtained from each participant. The Institutional Review Board (IRB) approved the study in accordance with the Helsinki Declaration and was approved by the Ethics Committee. (**IRB number**: 012098).

### Data collection

Preoperatively, we collected and analyzed the demographic characteristics of the patients, as well as the incidence of obesity-associated medical problems. The following parameters were recorded: operative time (OT), operative complications, length of hospital stay, and postoperative pain scores (using the Visual Analog Score [VAS]). Postoperative weight loss was expressed as a percent excess weight loss (%EWL), BMI reduction (BMIL), and percentage excess BMI loss (%EBMIL). Patients' scar satisfaction was assessed using the Patient Scar Assessment Questionnaire (PSAQ), and they were asked to grade their overall satisfaction with scar appearance as very satisfied, satisfied, or dissatisfied [11, 13]. The PSAQ comprises four validated subscales: appearance, consciousness, satisfaction with appearance, and satisfaction with symptoms. Each subscale includes a set of items with 4-point responses, scoring 1–4 points (1 point assigned to the most favorable category and 4 assigned to the least favorable).

### Surgical technique

BLOGB: All surgical procedures were conducted by the same surgeon (the first author) following a standardized perioperative protocol and operative technique [11, 14]. Patients were positioned in a modified split-leg position with a smaller angle of splitting. Straps and draping were placed at a lower level, exposing the lower abdomen. A closed pneumoperitoneum was established using an optical trocar insertion through the umbilicus.

**Fig. 1:**
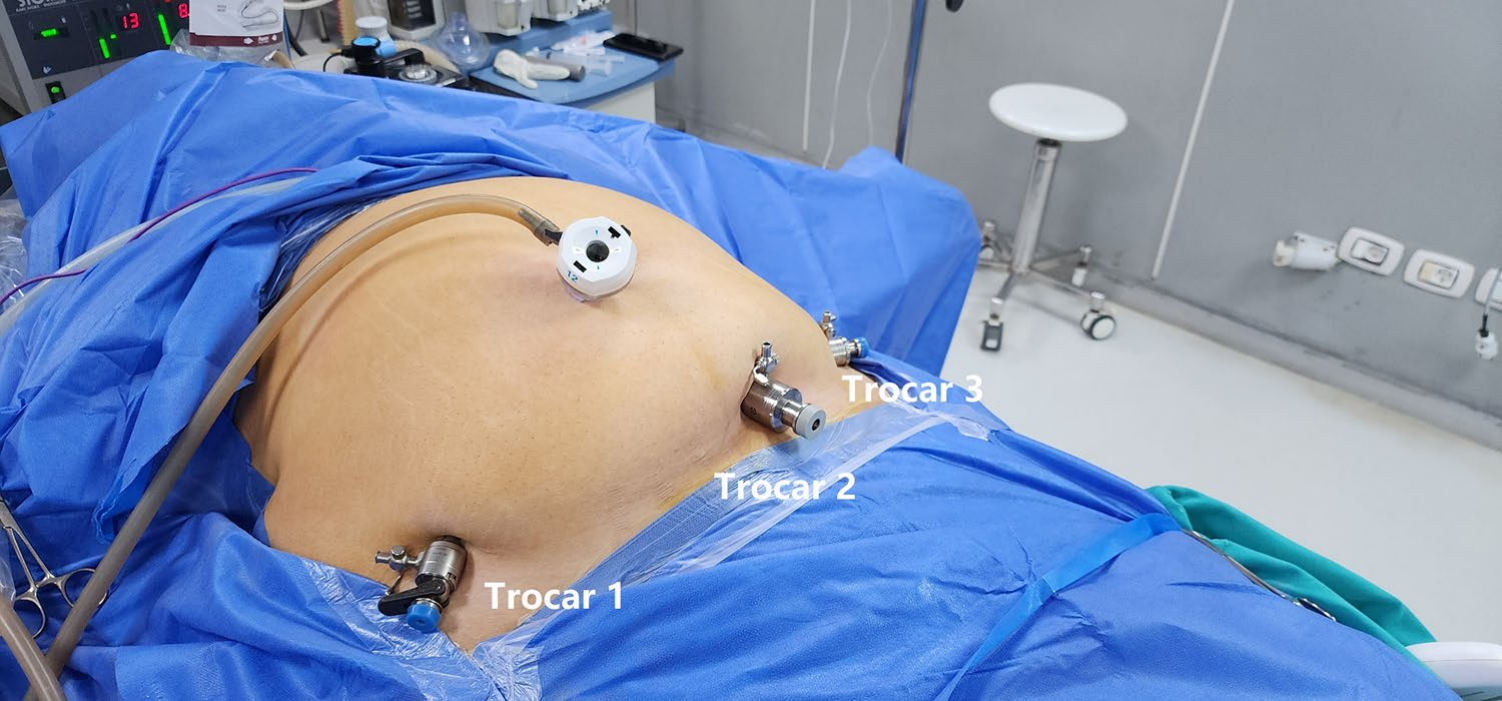
Trocars sites at the bikini line and umbilicus

Initial exploration was performed to rule out occult hiatal hernia and to check for major adhesions in the lower abdomen and pelvis. Under visual guidance, three trocars were then inserted along the bikini line: a 10 mm trocar was inserted to the left of the midline and used for camera insertion, and two 5 mm trocars were placed on the right and left sides of the bikini line (Fig. 1).

**Fig. 2:**
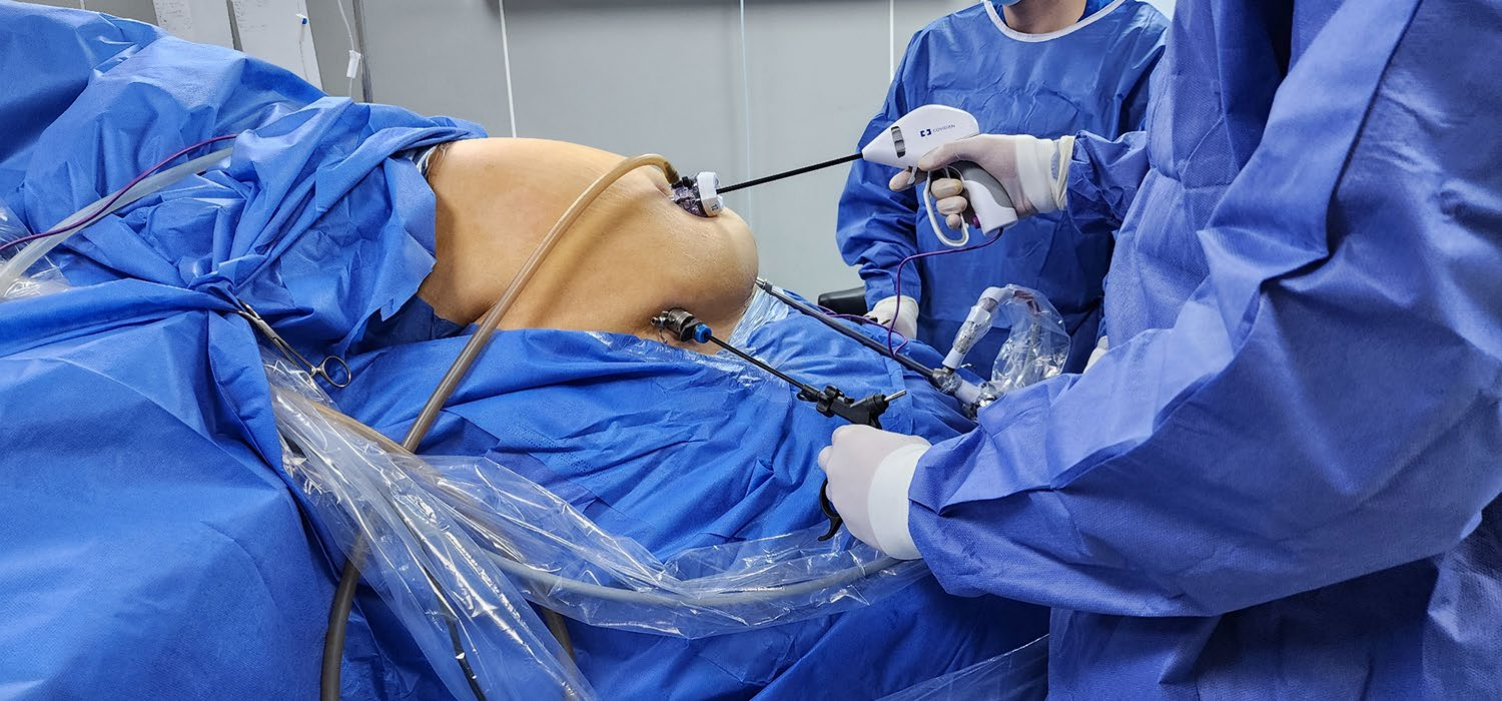
Intraoperative view demonstrating laparoscopic instruments

The surgeon stood on the right side of the patient, the cameraman between the patient's legs, and the first assistant on the left side of the patient. The surgeon used the umbilical and lower right 5-mm trocars for the right and left working hands, respectively, while the first assistant used the lower left 5-mm port (Fig. 2).

Bariatric laparoscopic instruments and equipment used included: 43 cm graspers and needle drivers, a 42 cm camera telescope (45°), endoscopic staplers, and a 44 cm bipolar energy source.

The surgeon uses the right lower bikini line trocar (Trocar 1) to hold the stomach and lift the liver simultaneously. Additionally, the assistant can also lift the liver from Trocar 3 whenever necessary. The surgeon uses the right lower bikini line trocar (Trocar 1) to hold the stomach and lift the liver simultaneously. Additionally, the assistant can also lift the liver from Trocar 3 whenever necessary.

The initial step of the procedure involved elevating the greater omentum to explore the anatomical position of the duodenojejunal junction and detect any anomalies. The dissection of the lesser omentum began near the lesser curvature of the stomach. After creating a window just distal to the Crow's foot, an initial stapler was fired transversely, followed by vertical stapling extended to the Angle of His. For this approach, we used the umbilical trocar for all stapler fires. With sufficient articulation, the first fire was initiated at the lesser curve. Additionally, the great mobility of the stomach enabled the correct positioning of the stapler. Throughout the entire staple line, 60 mm blue stapler reloads (3.5 mm). were used. A gastrojejunostomy was then created 180 cm from the ligament of Treitz using a blue reload. Closure of the ostomy was completed using single-layer 3/0 PDS sutures. A hypnotic stitch was added between the afferent limb and the gastric pouch, and an anti-twist stitch was placed between the efferent limb and the remnant stomach antrum. All skin incisions were closed using absorbable monofilament 3/0 sutures.

OAGB Conventional Technique [5, 7]: The procedure was performed as described above, with the main difference being the site of port placement [5]. A 5-trocar technique was used: a subxiphoid trocar for liver retraction, two 10 mm trocars inserted in the right and left mid-clavicular lines, a camera port placed to the left of the midline 10–15 cm below the xiphoid process, and a final 5 mm trocar inserted in the left anterior axillary line for the first assistant. The surgeon stood between the patient's legs, the cameraman stood on the right side of the patient, and the first assistant stood on the left side of the patient.

### Statistical analysis

Analysis was conducted using IBM SPSS software version 20.0. Categorical data were presented as numbers and percentages. The chi-square test was employed to compare between two groups. Alternatively, the Monte Carlo and Fisher Exact correction tests were utilized when more than 20% of the cells had an expected count of less than 5. For continuous data, normality was assessed using the Kolmogorov–Smirnov test. Quantitative data were expressed as range (minimum and maximum), mean, standard deviation, and median. The Student *t*-test was applied to compare two groups for normally distributed quantitative variables. Conversely, the Mann–Whitney test was used to compare two groups for quantitative variables that were not normally distributed. The significance of the obtained results was evaluated at the 5% level.

## Results

### Demographic and baseline characteristics

The present study included a total of 72 participants, with forty-two undergoing BLOGB and the remaining thirty undergoing standard OAGB.. The mean age of all studied patients was 41 ± 5.96 (range: 29.0–60.0), and 79.2%) were female. The mean preoperative BMI of the entire cohort was 40.01 ± 2.84 (range 32.60–44.44). Table 1 provides the demographic characteristics and prevalence of obesity-associated medical problems for all subjects. No significant differences were observed between the two groups in terms of age, sex, body mass index (BMI), or associated medical problems. All participants attended follow-up visits at 10 days, and 6 months. However, the follow-up rate at one, three and 12 months varied between 71.2% and 92.8%.

**Table 1:** Baseline characteristics and prevalence of comorbidities

	Total (*n* = 72)	BLOGB (*n* = 42)	OAGB (*n* = 30)	*p* value
Sex				
Male	15(20.8%)	7 (16.7%)	8(26.7%)	*0.632*
Female	57(79.2%)	35 (83.3%)	22(73.3%)	
Age (years)				
Mean ± SD (range)	41 ± 5.96 (29.0–60.0)	40.90 ± 4.56 (34.0–56.0)	41.13 ± 7.58 (29.0–60.0)	*0.437*
BMI (kg/m^2^)				
Mean ± SD (range)	40.01 ± 2.84 (32.60–44.44)	39.81 ± 2.55 (33.33–44)	40.31 ± 3.24 (32.6–44.44)	*0.231*
Associated diseases: n (%)				
Hypertension	29(40.3%)	17 (40.5%)	12 (40%)	*1.000*
DM	20(27.5%)	11 (26.2%)	9(30.0%)	*0.560*
Hypothyroid	2(2.8%)	0 (0.0%)	2(6.6%)	*0.486*
OSA	1(1.4%)	0 (0.0%)	1(3.3%)	*0.521*

*BLOGB* Bikini Line One-anastomosis Gastric Bypass, *p* significance, *OAGB* One-anastomosis Gastric Bypass, *BMI* body mass index, *kg* kilogram, *n* number, (Data expressed as a percentage (%) or mean ± standard deviation)

### Surgical outcomes

Scars from previous lower abdominal surgeries (five Caesarean sections and one abdominoplasty) were observed in 6 (8.3%) of all participants. These scars did not pose any challenges during port placement. Furthermore, the initial exploration revealed no significant abdominal or pelvic adhesions, and there was no need to relocate trocars for any of the participants. The mean operative time, length of hospital stay, operative complications, and postoperative pain scores are presented in Table 2. No mortality or major complications were observed.

**Table 2:** Operative time, Hospital Stay, Complications, and Pain score

	TOTAL (*n* = 72)	BLOGB (*n* = 42)	OAGB (*n* = 30)	*p*
Mean Operative time (minutes)	105.42 ± 18.91	110.71 ± 17.72	98 ± 18.27	0.002
	(range70–150)	(range90–150)	(range70–140)	
Mean hospital stay (days)	1.21 ± 0.39	1.19 ± 0.53	1.17 ± 0.39	0.762
	(range1–2)	(range1–2)	(range1–2)	
Leak *n* (%)	0 (0%)	0 (0%)	0 (0%)	
Bleeding	0 (0%)	0 (0%)	0 (0%)	
Surgical site infection	1 (1.4%)	0 (0%)	1(3.33%)	0.681
Postoperative pain score	2.93 ± 1.12	2.76 ± 1.01	3.43 ± 1.13	0.006
(VAS):	(range2–6)	(range2–4)	(range2–6)	

*BLOGB* Bikini Line One-Anastomosis Gastric Bypass, *OAGB* One-Anastomosis Gastric Bypass, *n* number, *p* significance, *SS* surgical site, (Data expressed as a percentage (%) or mean ± standard deviation)

### Scar appearance

Patient satisfaction with their scars, assessed through PSAQ scores and overall satisfaction grading during postoperative follow-up visits, is outlined in Tables 3, 4. The BLOGB group demonstrated significantly better scores and overall satisfaction.

**Table 3:** Patients scar assessment questionnaire (PSAQ) and score results

	BLOGB	OAGB	*p*
Scores range	(16.0–99.0)	(18.0–109.0)	
10 days	37.9 ± 3.32	41 ± 3.40	< 0.001^*^
1 month	26.2 ± 2.97	33.1 ± 4.07	< 0.001^*^
3 months	17.3 ± 1.02	23.2 ± 3.62	< 0.001^*^
6 months	16.8 ± 0.69	19.9 ± 2.03	< 0.001^*^

*BLOGB* Bikini Line One-anastomosis Gastric Bypass, *OAGB* One-Anastomosis Gastric Bypass, n number, p^*^ significance, (Data expressed as mean ± standard deviation)

**Table 4:** The overall satisfaction with scar appearance

	BLOGB	OAGB	*p*
10 days	(*n* = 42)	(*n* = 30)	
Very satisfied	38 (90.5%)	7 (23.3%)	< 0.001^*^
Just Satisfied	4 (9.5%)	23 (76.6%)	
1 months	(*n* = 38)	(*n* = 27)	
Very satisfied	34 (89.5%)	6 (22.2%)	< 0.001^*^
Just Satisfied	4 (10.5%)	21 (77.8%)	
3 months	(*n* = 32)	(*n* = 22)	
Very satisfied	28 (87.5%)	4 (18.2%)	< 0.001^*^
Just Satisfied	4 (12.5%)	18 (81.8%)	
6 months	(*n* = 42)	(*n* = 30)	
Very satisfied	42 (100%)	17 (56.7%)	0.005^*^
Just Satisfied	0 (0%)	13 (43.3%)	

*BLOGB* Bikini Line One-anastomosis Gastric Bypass, *OAGB* One-anastomosis Gastric Bypass, *n* number, p^*^ significance

### Outcomes of weight loss and obesity-associated medical problems

Postoperative percentages of excess weight loss (%EWL), BMI loss (%BMIL), and percentage of excess BMI loss (%EBMIL), as well as the resolution or improvement of obesity-associated medical problems during the follow-up visits, showed no significant differences between the two groups, as illustrated in Table 5.

**Table 5:** Weight loss and resolution or improvement of associated diseases

	BLOGB	OAGB	*p* value
%EWL 3 m 6 m 12 m	46.16% ± 4.52 (*n* = 32) 73.14% ± 4.85(*n* = 42) 90.88% ± 7.90(*n* = 39)	47.68% ± 5.08 (*n* = 22) 71.83% ± 5.32 (*n* = 30) 91% ± 7.11(*n* = 28)	0.126 0.142 0.474
%BMIL 3 m 6 m 12 m	20.91% ± 2.75 (*n* = 32) 332.14% ± 3.18(*n* = 42) 40% ± 3.04 (*n* = 39)	19.95% ± 2.75 (*n* = 22) 32.43% ± 3.77 (*n* = 30) 39.82% ± 2.45 (*n* = 28)	0.141 0.362 0.398
%EBMIL 3 m 6 m 12 m	43.97% ± 3.29 (*n* = 32) 68.52% ± 8.82 (*n* = 42) 87.36% ± 8.90 (*n* = 39)	42.91% ± 2.76 (*n* = 22) 70.87% ± 5.41 (*n* = 30) 88.111% ± 4.75 (*n* = 28)	0.110 0.101 0.342
T2DM (*n*) 1 m 6 m 12 m	*n* = 11 7(63.6%) 11(100%) 11(100%)	*n* = 9 7(77.7%) 8(8.88%) 9(100%)	0.449 0.883 0.878
Hypertension (*n*) 1 m 6 m 12 m	*n* = 17 7(41.23%) 10(58.8%) 10(58.8%)	*n* = 12 5(41.7%) 7(58.3%) 8(66.6%)	0.697 0.938 0.929
Hypothyroidism 6 m(*n* = 2)	–	2(100%)	
OSA 6 m (*n* = 1)	–	1(100%)	

*%EWL* percent Excess weight loss, *%BMIl* percentage body mass index lost, *%EBMIL* percentage excess body mass index loss, *T2DM* Diabetes Mellitus, *OSA* Obstructed sleep apnea, *n* number, *OAGB* One-anastomosis gastric bypass, *BLOGB* Bikini line one-anastomosis gastric bypass

## Discussion

In recent decades, surgeons have strived to improve the aesthetic outcomes of bariatric surgery through various techniques, including single-incision laparoscopic surgery (SILS). This method has been notably implemented in sleeve gastrectomy (SG) and Roux-en-Y gastric bypass (RYGB) procedures. Saber et al. and Huang et al. conducted case series studies on the SILS trans-umbilical approach in SG to minimize scar appearance and improve patient satisfaction rates, with favorable results reported [15, 16]. Additionally, the bikini line approach, described in sleeve gastrectomy and hiatal hernia repair (BLHHR) by the first author, demonstrated that this method is feasible, safe, and associated with less postoperative pain and high patient satisfaction with scar appearance [11, 12]. Consequently, we implemented the bikini line approach in one-anastomosis gastric bypass (OAGB); this technique had not been previously reported.

Forty-two out of 72 participants in the present study underwent BLOGB, while 30 patients underwent standard OAGB. Baseline characteristics showed no significant differences between the two groups regarding age, sex, BMI, and incidence of obesity-associated medical problems. Most of the study participants were female (79.2%), reflecting the current trend of higher prevalence of metabolic/bariatric surgery among women [17]. Both groups showed no significant differences in operative complications, hospital stay, weight loss, or resolution of obesity-associated diseases. However, the BLOGB patients had a longer mean operative time. They experienced less postoperative pain and reported greater satisfaction with the appearance of their scars.

The main challenge in applying this approach to perform a One-Anastomosis Gastric Bypass (OAGB) was primarily due to the placement of the ports through the lower abdomen, at a distance from the esophagogastric junction (EGJ). The use of long laparoscopic instruments and equipment allowed for convenient access to the EGJ with clear exposure and visualization of the hiatus. This facilitated the creation of an optimal-length gastric pouch and gastro-jejunal anastomosis. The low position of trocars at the bikini line did not hinder liver retraction or the placement of the first stapler on the lesser curve for pouch tailoring. The surgeon used the right lower bikini line trocar (Trocar 1) to hold the stomach and lift the liver simultaneously while using the umbilical trocar for all stapler fires. With sufficient articulation, the first staple was initiated at the lesser curve, and the great mobility of the stomach enabled the correct positioning of the stapler.

Moreover, the low placement of ports allowed direct access to the intestines, making intestinal measurement and the selection of a suitable jejunal loop length easier. Instrument maneuverability was adequate, and ergonomics were maintained throughout the procedure by ensuring an acceptable degree of spacing between trocars and optimal instrument triangulation (Figs. 1, 2). These findings confirm our previously reported results during BLSG and BLHHR [11, 12].

Scars from previous lower abdominal surgeries were observed in 5 (11.9%) of the BLOGB patients. These scars posed no difficulty during port placement at the bikini line. Initially, when designing this approach for sleeve gastrectomy, there were concerns that previous Caesarean sections might hinder port insertion due to underlying adhesions. However, it was found that most adhesions were minor, confined to the pelvis, and port placement was uneventful [11].

In the present study, the mean operative time (OT) for BLOGB patients was longer than that for those undergoing standard OAGB (110.71 ± 17.72 min vs 98 ± 18.27 min, *p* = 0.002), which may reflect the need for a longer learning curve. However, we believe that eventually, the OT will become comparable to that of the standard OAGB, as was the case following our experience with the bikini line sleeve gastrectomy [11]. Additionally, there was no significant difference in the length of hospital stay between the two groups (1–2 days, *p* = 0.762). Both the operative time and hospital stay for all participants were within acceptable ranges. Large patient cohort trials have reported a mean OT ranging from 86 to 110 min [18]. Similarly, the average length of stay for OAGB patients was previously reported to range from 1 to 5 days, with longer stays observed in patients with higher BMI and those undergoing the surgery as a revision [18–20]. The ASMBS has recognized OAGB as a procedure with a relatively short operative time and low complication rates [21].

Reduced postoperative pain and improved aesthetic outcomes are important concerns following surgical procedures. In the present study, the mean postoperative pain score following BLOGB was lower compared to the standard approach, possibly because lower abdominal scars tend to cause less postoperative pain than upper abdominal scars. Moreover, in the BLOGB group, the Patient Scar Assessment Questionnaire (PSAQ) demonstrated gradual improvement in scores, reaching optimal (lowest) scores by the 6-month follow-up visit. All patients expressed increased satisfaction with their scar appearance; 87.5% and 100% reported feeling very satisfied at 3 and 6 months post-surgery, respectively. In contrast, only 18.2% and 81.8% of OAGB patients expressed similar levels of satisfaction. The placement of scars below the abdominal folds rendered them inconspicuous, positively influencing scores, boosting satisfaction levels, and potentially having a positive psychological impact, thereby enhancing the overall quality of life. Another appealing aspect of this approach is the possibility of removing the scars during any future abdominoplasty, potentially making the procedure relatively scar-free. Aesthetic improvement is an important concern, especially among young women undergoing bariatric surgery. Another appealing aspect of this approach is the possibility of removing the scars during any future abdominoplasty, potentially making the procedure relatively scar-free.

No mortality or major complications were observed in the present study; only one patient in the OAGB group developed a surgical site infection (SSI). One might have anticipated a higher rate of port site infection in the BLOGB group, considering the low position of the scars within the abdominal folds. Although we did not observe leaks or hemorrhage in any participants, the potential for these complications in the early postoperative period following OAGB cannot be ruled out [5]. The incidence of leaks has previously been reported to range from 0.1% to 1.9%, and bleeding from the staple line has been reported in less than 3% of cases in OAGB [5, 21, 22]. Due to the shorter operative time and simplicity of the surgical technique, OAGB has shown fewer early and late surgical complications, ranging from 4% to 7.5% [21].

The mean percentage of excess weight loss (%EWL) and decrease in BMI observed during the follow-up visits were satisfactory and did not differ significantly between the two groups. The highest weight loss was noted at 12 months following surgery (90.88 ± 7.90% and 91 ± 7.11%, p = 0.474). These results are comparable to those reported in previous studies, which demonstrated outstanding weight loss outcomes following primary OAGB [5, 21, 23, 24].

Resolution or improvement of obesity-associated medical problems was observed in all participants, with no significant differences between the two groups. Improvement or remission started early, from the first postoperative month. At 12 months following surgery, resolution of T2DM was observed in all patients. Improvement in hypertension varied between 41.2% and 66.6% of the patients depending on the time interval following surgery. These results are comparable to those previously reported. Complete resolution or substantial improvement in obesity-associated medical problems following OAGB, including type 2 diabetes mellitus (T2DM), insulin resistance, hypertension, hyperlipidemia, liver steatosis, and obstructive sleep apnea, has been previously demonstrated [22, 24]. Lee et al. reported a 100% resolution in patients with metabolic syndrome at 2 years [20].

## Strength and limitation

This is the first study to report the outcomes of Bikini line access in patients undergoing OAGB. The main limitations of this study are the relatively small number of patients who underwent BLOGB and the short follow-up period, which was less than 12 months for most participants.

## Conclusion

BLOGB was potentially feasible, safe, and effective, with improved aesthetic outcomes and reduced postoperative pain. There were no significant differences in weight loss, operative complications, or the resolution of obesity-related medical issues compared to standard OAGB. However, the mean operative time for BLOGB was longer, suggesting a potentially steep learning curve. It may be suitable for a select group of patients concerned with scar appearance. This study was descriptive and pilot in nature. Large-scale prospective controlled studies are necessary to assess the benefits and risks of this approach, ensuring that safety is not compromised while pursuing aesthetic benefits.
